# Ginsenosides and Tumors: A Comprehensive and Visualized Analysis of Research Hotspots and Antitumor Mechanisms

**DOI:** 10.7150/jca.88783

**Published:** 2024-01-01

**Authors:** Demeng Xia, Shuo Wang, Kaiwen Wu, Na Li, Wei Fan

**Affiliations:** 1 Department of Pharmacy, Seventh People's Hospital of Shanghai University of Traditional Chinese Medicine, Shanghai 200120, China.; 2 Department of Clinical Medicine, Hainan Health Vocational College, Hainan 572000, China.; 3 Department of Clinical Laboratory. Naval Hospital of Eastern Theater of PLA, Zhoushan, Zhejiang Province 316000, China.; 4 Department of Gastroenterology, The Third People's Hospital of Chengdu, The Affiliated Hospital of Southwest Jiaotong University, Chengdu, Sichuan 610031, China.; 5 School of Medicine, Shanghai University, Shanghai 200444, China.

**Keywords:** bibliometrics, chemotherapy, ginsenoside, hotspots, tumor.

## Abstract

**Background:** Ginsenoside, the main active constituent of traditional Chinese medicine Ginseng, has been shown to play an important role in the prevention and treatment of cancer. However, the literature as well as the antitumor mechanisms of ginsenosides has not yet been systematically studied.

**Methods:** We screened all relevant literature on ginsenosides and tumors from Web of Science during 2001-2021 and analyzed the extracted terms of these publications by VOSviewer and CiteSpace. DAVID online tool was used to perform Gene Ontology enrichment analysis and Kyoto Encyclopedia of Genes and Genomes pathways analysis of ginsenoside-related genes. Cytoscape and String software were used to construct the interaction networks of ginsenoside-related genes and corresponding proteins.

**Results:** A total of 919 publications were included in the study. A total of 122 identified keywords were mainly divided into 3 clusters: “pharmacological function research”, “functional validation in animal models” and “anti-tumor efficacy and mechanism”. The keywords of “oxidative stress” had the strongest citation burst in the past 5 years. A total of 50 genes were identified as ginsenoside-related genes in tumors. They have the function of regulating gene expression and apoptosis, and they are closely related to signaling pathways in cancers. Ginsenoside-related genes form a complex interactional network, in which TP53 and IL-6 are centrally located.

**Conclusions:** We explored and revealed research hotspots related to the ginsenosides and tumors. More precise anti-tumor mechanism research will be promising in the future. TP53 and IL-6 may be the key points to comprehending the anti-tumor mechanism of ginsenosides.

## Introduction

Ginseng, the root and rhizome of Panax ginseng C.A. Meyer (Araliaceae), displays versatile roles in the treatment and prevention of numerous diseases, and has been used as a traditional Chinese medicine for thousands of years. With the technological progress of separation, purification and chemical analysis, such as Liquid chromatography coupled with tandem mass spectrometry (LC-MS), the research focus has also shifted from the original ginseng saponin mixture to various monomers extracted from ginseng 1. Among the compounds researched in ginseng plants, ginsenoside is regarded as the main bioactive component 2. The basic chemical structure of ginsenosides includes the fundamental skeleton and the linked sugar moieties 3. The fundamental skeleton, also named as sapogenin, includes three types of protopanaxadiol (PPD), protopanaxatriol (PPT), oleanolic acid (OA), and C17 side-chain varied (C17SCV) subtypes. PPD and PPT belong to classical tetracyclic triterpene, also is the earliest recognized ginsenoside skeleton. The C17SCV subtype has a similar fundamental skeleton to that of PPD and PPT, also belonging to tetracyclic triterpene. As chemical analysis became more precise, variations on the C17 side-chain of C17SCV subtypes were uncovered, comprising of H_2_O-addition, hydroxylation, peroxidization, carbonylation, dehydrogenation, cyclization, oxidation, and degradation, etc. The C17SCV subtype can be as the artifacts in the steaming process of the root, or ginsenosides extracted from the flowers, leaves or fruits of ginseng plants 4, 5. In contrast to other types, oleanolic acid belongs to pentacyclic triterpene. As for the sugar moieties, also named as glycosyl units, ginsenosides are different from each other in the type, number and linkage position at the fundamental skeleton 6.

Tumorigenesis is a complex multistep process, which is not only composed of the unlimited proliferation of tumor cells, but also the heterotypic interactions of multiple cell types. Tumor cells even recruit normal cells to create a suitable microenvironment for tumor progression 7. Because current treatments of tumors have met with moderate success, novel approaches to overcome tumors are required. Although the optimal recommended dosage is not completely consistent, several clinical studies have supported the benefits of ginseng using. Use of ginseng improves cancer-related fatigue and overall quality of life, and have a greater effect on NSCLC patients' immune function. Plenty of articles have reported that ginsenosides have a wide variety of anti-tumor activities. For instance, ginsenoside Rg3 could inhibit the proliferation of human colorectal cancer cell lines by inhibiting the C/EBPβ/NF-κB signaling pathway in a dose-depended manner 8. Ginsenoside Rg5 could trigger caspase-dependent apoptosis and autophagy to exert a pro-death effect by suppressing the PI3K/Akt signaling pathway in breast cancer cells 9. Ginsenoside Rh2 demonstrates potent killing capability of colorectal cancer cell by inducing caspase-dependent apoptosis and the caspase-independent paraptosis at the same time 10. Ginsenoside Rk3 could exert anticancer effects via inhibiting proliferation, inducing apoptosis and inhibiting angiogenesis for non-small cell lung cancer (NSCLC) cells both *in vitro* and *in vivo* 11. In addition to being a new promising therapeutic agent because of antitumor activity, ginsenosides could also be combined with chemotherapeutic drugs to alleviate the side effects and multidrug resistance (MDR). Ginsenoside derivative Rp1 could modulate transporter-based classical MDR by decreasing MDR-1 activity to inhibit effluxes of anticancer drugs, and synergistically induced cell death with anti-cancer drugs in drug-resistant cell lines 12. The beneficial effects of ginsenoside Rb3 on renal damage might closely relate to the inhibition of ROS-induced apoptosis and autophagy through AMPK/mTOR signaling pathways. Besides, Ginsenoside Rb3 could alleviate cisplatin-induced nephrotoxicity partly through regulation of AMPK-/mTOR-mediated autophagy in human cell lines and ICR mouse models 13. Although numerous articles involving various biological activities on ginsenosides and tumors have been reported, the literature in this field and the pharmacological mechanism has not yet been systematically studied.

**Figure 1:**
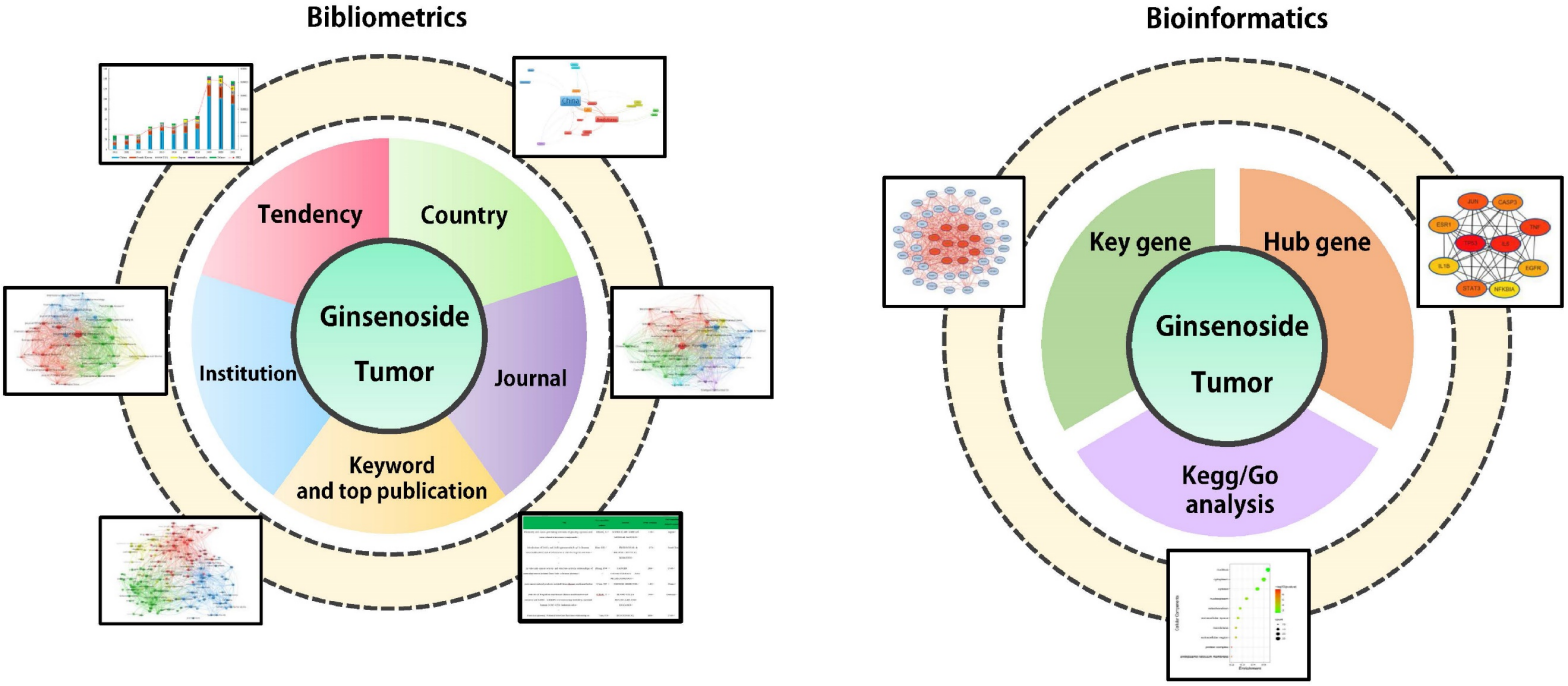
Flow chart of research approach of this study. A total of 919 papers and 50 ginsenoside-related genes were downloaded from Web of Science and GeneCards database between 2001 and 2021 respectively. We performed bibliometric analysis for final-included papers, including global contributions and field activity, collaboration between countries, institution and journal distribution, as well as Co-occurrence analysis of key words. We performed bioinformatics analysis for ginsenoside-related genes, including Gene Ontology (GO) enrichment analysis, Kyoto Encyclopedia of Genes and Genomes (KEGG) pathways analysis and protein-protein interaction (PPI) network analysis.

Bibliometrics analyzes publications quantitatively through mathematical and statistical methods, helping to depict the knowledge structure, research context and content distribution of a certain field 14. Bibliometric analysis could implement comparisons between different countries, institutions and journals to mine collaborative relationships within specific field. Keywords represent the main topic of publications. Through cluster analysis and co-occurrence analysis of article keywords, bibliometrics is conducive to systematically understanding the research status and consequently deciphering the hot topics in this field 15. Compared to traditional review, bibliometrics offers distinct quantitative attributes and extensive coverage. It presents overarching trends, evolving developments, and correlated relationships within research through statistical analyses, visualizations, and other methods. Bibliometrics enables a more objective and comprehensive understanding of research domains, furnishing a scientific foundation and informed support for academic research, policy formulation, and scientific management. Bioinformatics develops methods and software tools to process biological data to obtain information and make predictions. After obtaining genetic data, bioinformatics could construct action networks of related genes through gene ontology (GO) enrichment analysis, Kyoto Encyclopedia of Genes and Genomes (KEGG) pathways analysis and protein-protein interaction (PPI) analysis, which helps to macroscopically display characteristics of related genes and predict potential mechanisms 16, 17. In this study, we collected publications about ginsenosides and tumors in the Web of Science (WOS) database during 2001-2021. We also obtained related genes of ginsenosides and tumors from GeneCards and NCBI database. Bibliometric tools are employed to analyze collaborative relationships and article keywords, and bioinformatics tools are used for constructing pharmacological network of related genes and corresponding proteins, respectively. The workflow of this study is displayed in **Figure 1**. We aim to map thematic trends, elucidate mechanisms and predict hotspots related to the ginsenosides and tumors, providing a reference for future research.

## Material and Methods

### Data acquisition and search strategy

**Figure 2:**
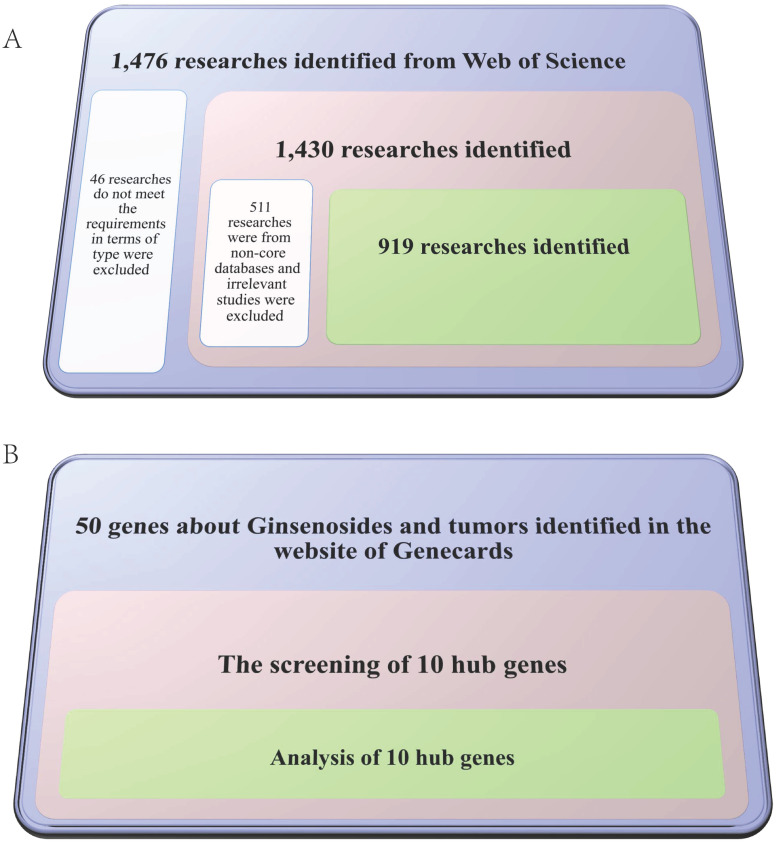
Flow chart of the screening process for papers (Figure 2A) and ginsenoside-related genes (Figure 2B).

Web of Science (WOS) has widely collected world-class academic research results that have been widely used in bibliometric analysis. We selected WOS as a data source for a comprehensive search on ginsenosides and tumors from 2001 to 2021. The search strategies were as follows: TS = Ginsenoside and (tumor OR tumour OR cancer OR neoplasia OR neoplasm OR malignanc* OR carcinoma OR adenocarcinoma OR oncology OR lymphoma OR leukemia OR melanoma). Authors (DMX and SW) independently screened and extracted all data from the final included articles, including titles, countries/regions, institutions, journals, dates of publication, keywords, citations, H-index, etc. The screening process is shown in **Figure 2A**.

GeneCards (http://www.genecards.org/) is a searchable and comprehensive database that integrates gene-centric data from approximately 150 web sources. The same search strategies as retrieving literature in WOS were employed to obtain ginsenoside-related genes in tumors in GeneCards database. We also screened out 10 hub genes to further highlight the most relevant genes, and the screening process is shown in **Figure 2B**. All data were obtained online, and no ethical certification was required. All searches were conducted on February 28th, 2022, to avoid bias related to database updates.

### Bibliometric analysis

Relative research interest (RRI) indicates the relative activity in a certain field, and RRI is defined by weighted publications per year (WPPY) and all weighted publications per year in PubMed (AWPPY). We used Excel to calculate the RRI and the country distribution of publications.

The visualization software programs VOSviewer and CiteSpace are popular in bibliometric analysis. For bibliographic items extracted from final included articles, we used VOSviewer software to perform coupling analysis of countries/regions and institutions 18. We used CiteSpace to analyze the centrality of institutions, and online platform bibliometric (https://bibliometric.com) to analyze the partnership between countries/regions 19. VOSviewer software is also used to perform co-citation analysis of journals, co-occurrence and clustering analysis of keywords. The average appearing year (AAY) was used to describe the relative novelty of keywords.

### Bioinformatics analysis

For ginsenoside-related genes, website tool DAVID (http://david.abcc.ncifcrf.gov/) was employed to conduct GO enrichment analysis and KEGG pathways analysis. The count means the number of genes included in ginsenoside-related genes. We selected the 50 terms with the highest count for display. We also screened out 10 hub genes for further study. STRING database (http://www.string-db.org/) was employed to construct protein-protein interaction (PPI) network. We also input the data of ginsenoside-related genes to Cytoscape software to analyze the PPI network.

## Results

### Global contributions and field activity

Depending on the inclusion criteria, 919 articles related to ginsenosides and tumors were included in the final analysis. China (203 publications) is the most productive country, followed by South Korea (215 publications), USA (86 publications), Japan (14 publications). Australia (14 publications) and Canada (14 publications) tied for 5th. The data of H-index was consistent with country distribution. The sum of citations is slightly different. The top 4 countries still are China (10395 citations), South Korea (5386 citations), the USA (3258 citations) and Japan (660 citations), and Canada (459 citations) ranks fifth **(Figure S1A)**.

In terms of the annual distribution, although there was a mild reduction in last 2 years, the overall trend of the publication output is increment. It is worth mentioning that during 2018-2019, there was a sharp increase in the publication output, mainly due to the contributions from China. The trend of the Relative research interest (RRI) was similar, suggesting that the field of ginsenosides and tumors is receiving more attention in general **(Figure S1B)**.

### Analysis of collaboration between countries

The collaborations between countries are shown in **Figure S2A**. The size of the node indicates the publication output, and the width of the connecting line indicates the degree of collaboration. China has the strongest total link strength, which means that China has a strong influence in this field. The same results were also obtained using the online platform bibliometric. The area of the sector represents the number of publications, and the connection represents the collaborative relationship. In addition to the huge volume of publications from China in this field, we can also observe the close collaboration between China and the USA.

Many countries have concentrated years of article output. The USA and South Korea had the most publications concentrated before 2016. Then articles from the China were mainly published after 2018. From the perspective of centrality, China is also the country with the highest centrality. Although the frequency of South Korea and the USA is relatively small, they also have important contributions in this field. Comprehensive analysis of mutual collaboration on a global scale, the China, South Korea and Japan constitute a research center in the field of ginsenosides and tumors, and the USA and Canada constitute another **(Figure S2B)**.

### Analysis of institution distribution

Jilin University made the most considerable contribution to the studies of ginsenosides and tumors, about 7.69% of the published papers. Seoul National University (4.84%) and Kyung Hee University (4.40%), Shenyang Pharmaceutical University (3.30%) Nanjing University of Chinese Medicine (3.08%) ranked second to fifth, respectively. Among the top 10 institutions by the number of papers, 7 institutions are located in China, 2 are located in South Korea, and 1 is in the USA **(Figure S3A)**.

Like the collaboration between different countries, there are close and complex collaborative relations between different institutions. According to the area of nodes and the width of connective lines, Jilin University has the strongest total link strength, which means that the Jilin University is regarded as pivotal institution in this field **(Figure S3B)**.

### Analysis of journal distribution

Different journals have different corresponding fields. Hence, we analyze the journal distribution of ginsenosides and tumors. The *Journal of Ginseng Research* (impact factor=6.06, 2021) published the most studies, which accounts for 5.5% of included publications. Followed by the journal *Molecules* (impact factor=4.411, 2021), which account for 2.53%. *Evidence-based Complementary and Alternative Medicine* (impact factor=2.629, 2021) and *Molecular Medicine Reports* (impact factor=2.952, 2021) are tied for third, accounting for 2.2%. And *Biomedicine & Pharmacotherapy* (impact factor=6.529, 2021) with 1.98% ranked fifth **(Figure S4A)**.

As a specialized journal for ginseng research, *Journal of Ginseng Research* is not only with the highest number of publications, but also has the highest total link strength, suggesting the most prominent journal **(Figure S4B)**. From a timeline perspective, publications from the *Journal of Ginseng Research* were intense after 2018, which suggests that the content of the journal is relatively novel **(Figure S4C)**.

### Co-occurrence analysis of key words

Keywords provide the summary of publications. Co-occurrence analysis of keywords helps to grasp the inner link between publications, and further cluster analysis is conducive to systematically understanding the current research status in this field. VOSviewer was employed to analyze high-frequency keywords (defined as appearing more than five times in titles and abstracts) in final-included publications. VOSviewer used clustering algorithms to group similar keywords into clusters. Based on the characteristics and composition of keyword clusters, a theme or research direction can be assigned to each cluster. A total of 122 identified keywords were mainly classified into three clusters: “pharmacological function research”, “functional validation in animal models” and “anti-tumor efficacy and mechanism”.

**Figure 3:**
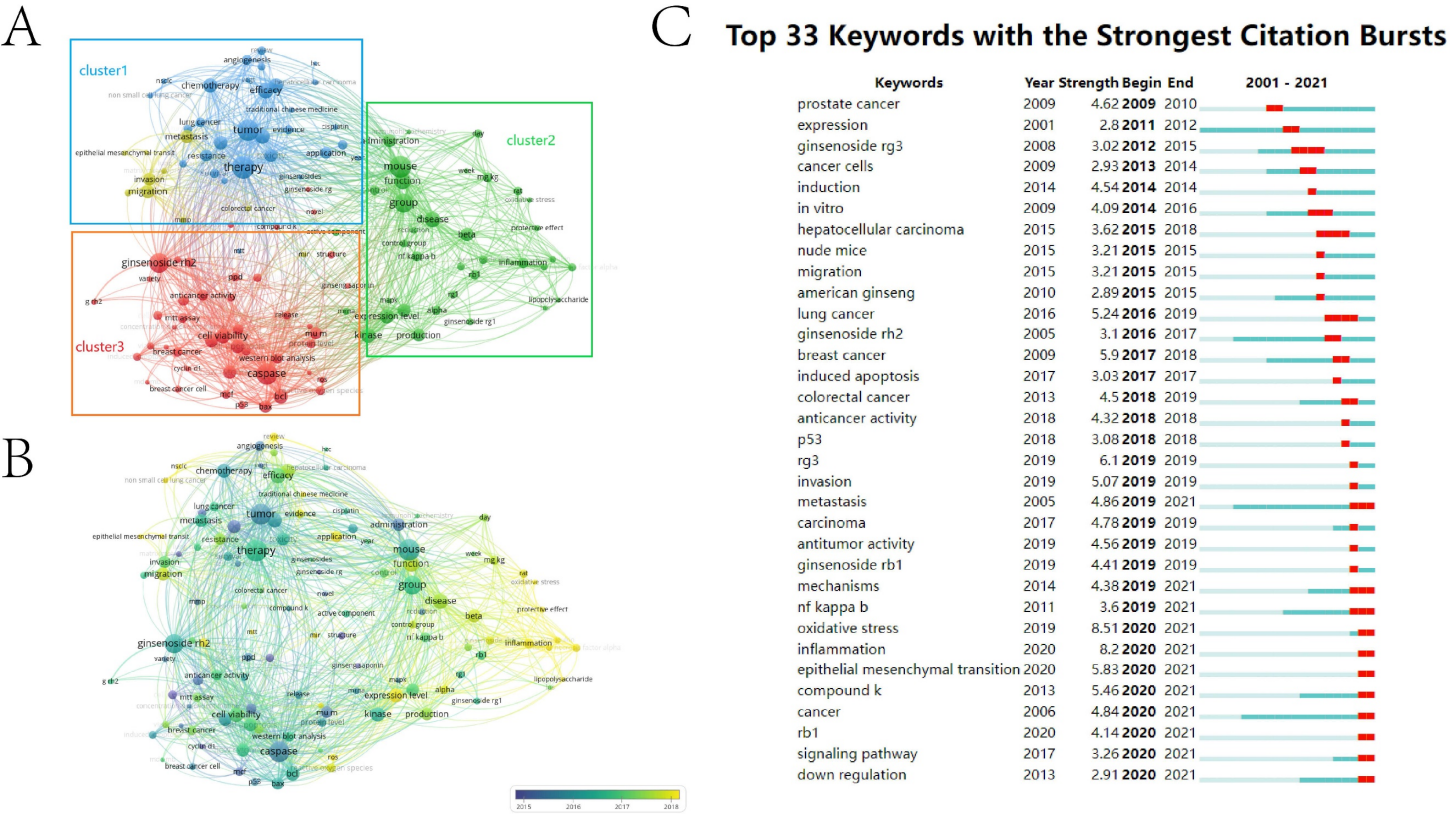
Mapping of keywords in studies on ginsenoside in tumour. (A) Clustering analysis of keywords based on VOSviewer. (B) Chronological order of keywords based on VOSviewer. (C) Keywords with the strongest citation bursts based on CiteSpace.

In the cluster of “pharmacological function research”, the keywords with the highest frequency were therapy (151 times), tumor (145 times), efficacy (101 times), combination (94 times) and chemotherapy (90 times). In the cluster of “functional validation in animal models”, the words were mouse (147 times), group (126 times), function (105 times), disease (102 times) and kinase (95 times). In the cluster of “anti-tumor efficacy and mechanism”, these words were caspase (141 times), ginsenoside rh2 (131 times), cell viability (94 times), bcl (89 times) and cell apoptosis (84 times) (**Figure 3A**). Chronological order of keywords is presented from dark blue to bright yellow. In the cluster of “pharmacological function research”, the earliest word was “tumor cell” (AAY 2015.24), with 50 times, and the latest word was “review” (AAY 2018.9091), with 50 times. In the cluster of “functional validation in animal models”, the earliest word was “administration” (AAY 2015.4304), with 79 times, and the latest word was “oxidative stress” (AAY 2019.1724), with 29 times. In the cluster of “anti-tumor efficacy and mechanism”, the earliest keyword was “ginseng saponin” (AAY 2011.8387), with 31 times, and the latest word was “ROS” (AAY 2018.1053), with 38 times. Tumors are strongly associated with chronic inflammation 20. According to the annual distribution, we found that the research hotspot on ginsenosides and tumors have shifted gradually from pharmacological function and mechanism exploration to validation in animal models and prevention of tumorigenesis in the stage of inflammation (**Figure 3B**).

The content of early research is more focused on anti-tumor efficacy and cytological effects, and the research hotspot has converted to animal experiments and exploring the mechanism in recent years. Top 33 keywords in the strongest citation burst reflects the same trend. Before 2019, the research mainly pays attention to cytological effects and anticancer activity *in vitro*. The most studied ginsenosides are “invasion” and “lung cancer”. Since 2019, the research focus on ginsenosides and tumors has gradually shifted to the specific cell biological processes, and molecular mechanisms are increasingly precise. In the past 5 years, keywords of “oxidative stress”, “inflammation” and “EMT” have experienced a sudden increase, which means the emergence of new hot spots in this field, among which keywords of “oxidative stress” had the strongest citation burst (**Figure 3C**).

### GO and KEGG analysis of ginsenosides-related genes

**Figure 4:**
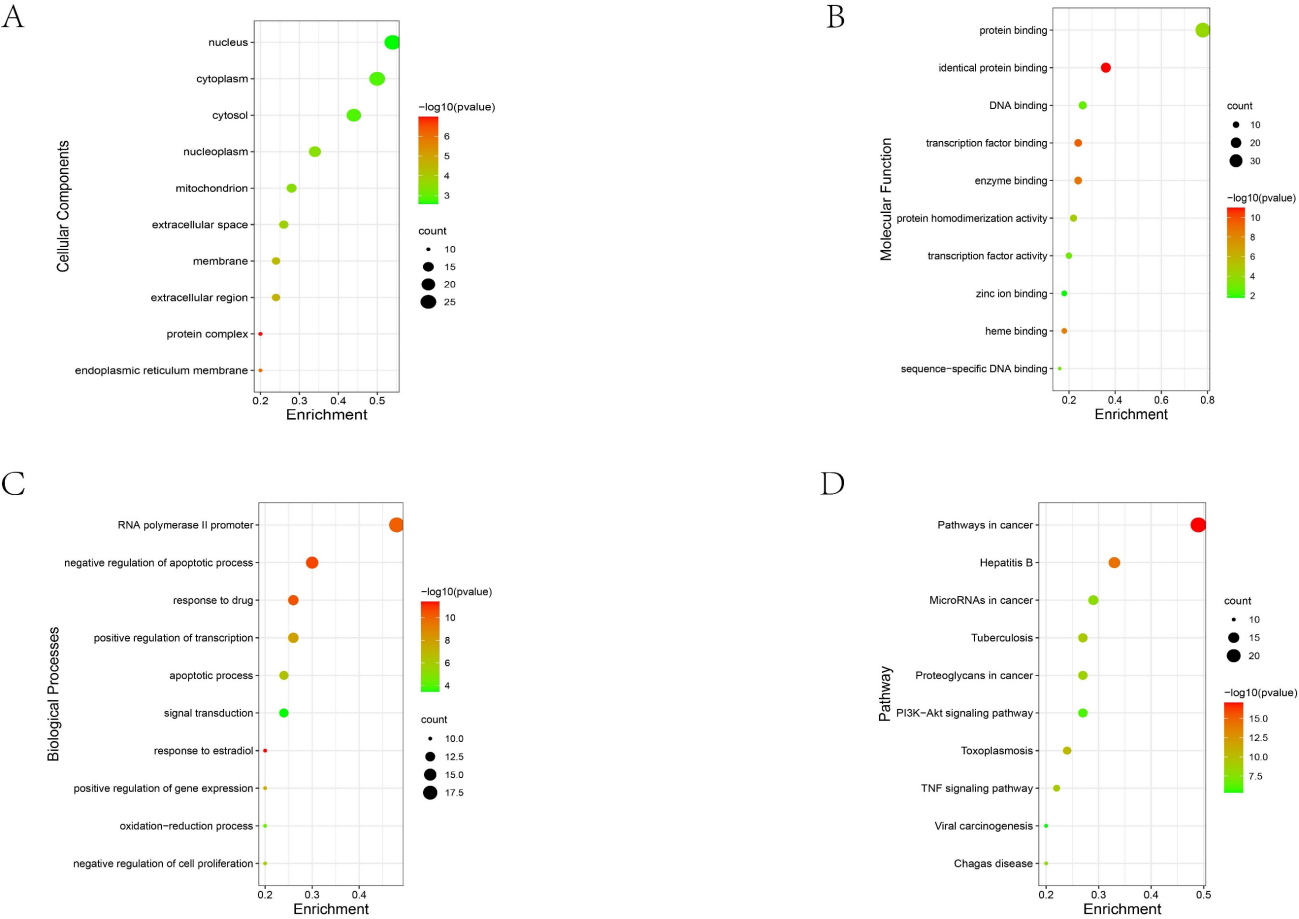
GO and KEGG analysis of ginsenosides-related genes. (A) The Cellular the Component analysis. (B) The Molecular Function analysis. (C) The Biological Process analysis. (D). The KEGG pathway analysis. The node size indicates the count of enriched ginsenosides-related genes, and the node colour indicates the P value.

To further systematically investigate the multiple mechanisms of ginsenosides and tumors, we obtained a total of 50 ginsenosides-related genes through GeneCards database. and then GO enrichment and KEGG pathway analysis were performed on them. The Cellular Component suggested that these genes are widely dispersed, and top5 terms were nucleus (GO:0005634), cytoplasm (GO:0005737), cytosol (GO:0005829), nucleoplasm (GO:0005654) and mitochondrion (GO:0005739), which suggest that these related genes are widely distributed in cells (**Figure 4A**). The main Molecular Function terms were protein binding (GO:0005515), identical protein binding (GO:0042802), DNA binding (GO:0003677), transcription factor binding (GO:0008134) and enzyme binding (GO:0019899), which suggested that binding is the main action mode of these genes (**Figure 4B**). The main Biological Process terms were positive regulation of transcription from RNA polymerase II promoter (GO:0045944), negative regulation of apoptotic process (GO:0043066), response to drug (GO:0042493), positive regulation of transcription, DNA-templated (GO:0045893) and apoptotic process (GO:0006915), mainly involving regulation of gene expression, apoptosis and drug susceptibility (**Figure 4C**).

In the KEGG pathway analysis, the most enriched term for these ginsenosides-related genes was Pathways in cancer (hsa05200), reflecting the broad roles in tumorigenesis. Hepatitis B (hsa05161), MicroRNAs in cancer (hsa05206), Tuberculosis (hsa05152) and Proteoglycans in cancer (hsa05205) ranked second to fifth, respectively (**Figure 4D**).

### Protein-protein interaction analysis of hub genes

**Figure 5:**
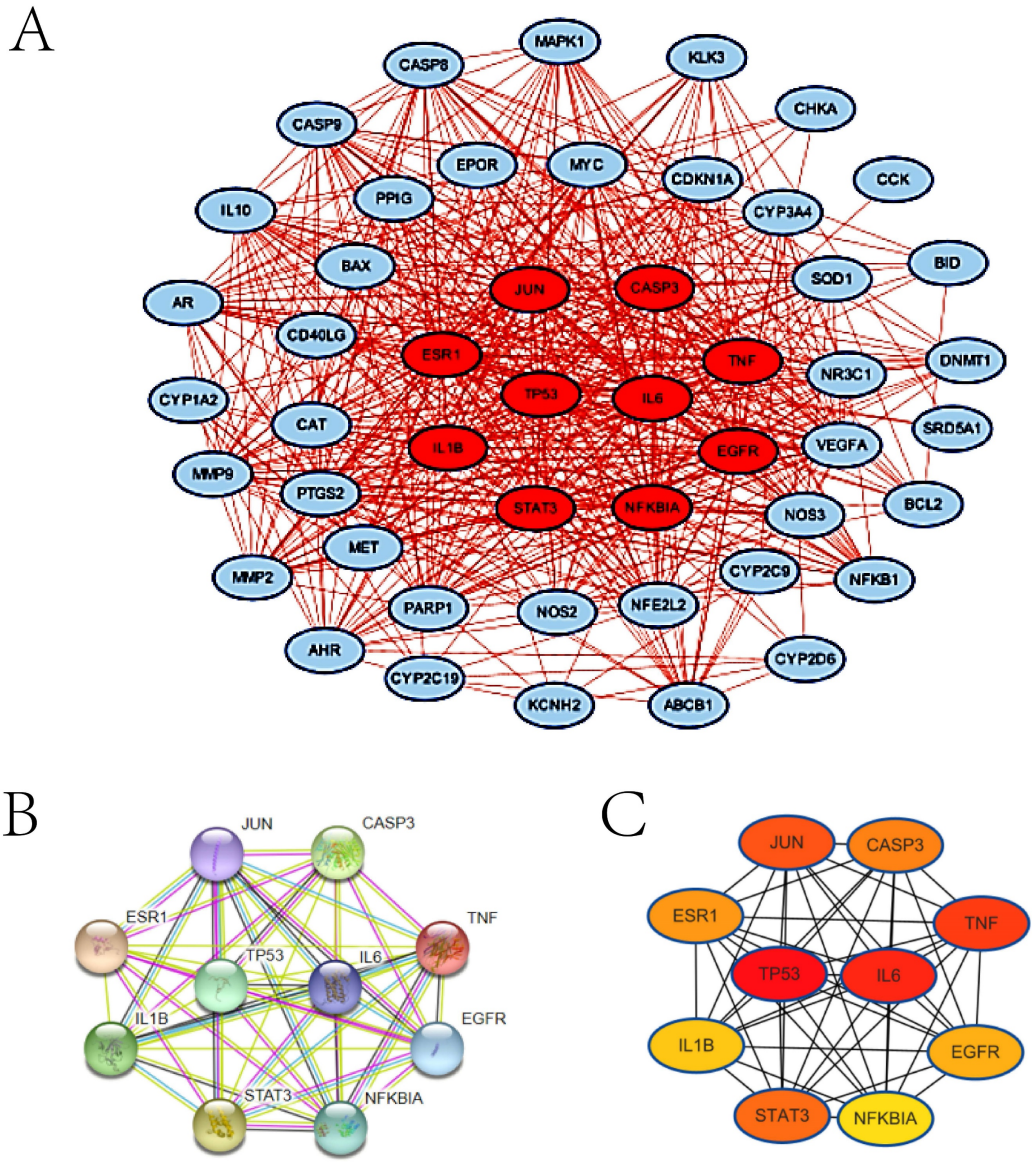
Protein-protein interaction (PPI) analysis of ginsenosides-related genes. (A) The PPI network of total 50 ginsenosides-related genes by STRING database. Each node represents a different protein, and the line connecting the node indicate a different action mode. (B) The PPI network of total 50 ginsenosides-related genes by Cytoscape software. The node colour indicates the total link strength. Red represents high and blue represents low. (C) The PPI network of 10 hub genes by STRING database. (D) The PPI network of 10 hub genes by Cytoscape software.

Considering that protein is the carrier of life activities, we then performed protein-protein interaction (PPI) analyzed by STRING database (**Figure 5A**) and Cytoscape software (**Figure 5B**). Ginsenoside-related genes form a complex network with each other, and there is more than one way of interacting with each other. In order to clarify the core of this network, we further stratified these genes according to the strength of gene interaction. Among them, top 10 genes are TP53, IL6, TNF, JUN, STAT3, CASP3, ESR1, EGFR, IL-1β and NFKBIA (consisting of hub genes), and then we reconstructed interaction network of hub genes (**Figure 5C**). TP53 and IL-6 are at the center of the network, implying that these two genes may be the key points for in-depth understanding of the mechanism of ginsenosides.

## Discussion

In this study, we systematically analyze the publications about ginsenosides and tumors, and bibliometric tools are employed to analyze collaborative relationships between countries/regions, institutions and journals. We also illustrate the research progress, research status and future trends in this field through the co-occurrence analysis of keywords. Besides, we summarize the potential mechanism of ginsenosides through bioinformatics analysis of ginsenoside-related genes. This study is beneficial to find milestone achievements and grasp future research hotspots.

### Two research centers are the main body of research in this field

With the progress of separation and modern analytical technology, the chemical properties and biological functions of ginsenosides have also been increasingly discovered, including the anti-tumor activity 21, 22. Countries with a high number of publications in the field of ginsenosides and tumors also happen to be the major producers of ginseng (**Figure S1**). Panax ginseng (known as Asian or Korean ginseng) and Panax quinquefolius (known as American ginseng) are the most commonly used type of ginseng. Panax ginseng grows or cultivated mainly in China and South Korea, and Panax quinquefolius grows or cultivated mainly in the USA and Canada 23. These 2 main production areas not only have strong influence in this field (**Figure S2A and S2B**), but also correspond to 2 research centers consisting of China-South Korea-Japan and the USA-Canada, respectively (**Figure S2D**). Academic institutions are the embodiment of scientific research strength of country. The top 10 institutions with the highest publication volume are all located in these 2 research centers (**Figure S4**). This phenomenon reflects that cooperation in this field are still limited to specific regions. More efforts are needed to promote the research and application of traditional Chinese medicine as well as purified extracts on a global scale.

From the perspective of the number of publications and RRI, there was a leap in 2018-2019 (**Figure S1**). China is the main factor in this leap, and articles from the China were mainly published after 2018, corresponding to this time point **(Figure S2B)**. Besides, the journals with the most publications *Journal of Ginseng Research* also responded to this time point (**Figure S3**). But the downward trend in 2020-2021 is considerable (**Figure S1**), indicating the slowdown in activity in this field. This trend also reflected in the chronological order of keywords. In the cluster of “pharmacological function research”, the latest word was “review” (AAY 2018.9091), which suggests lack of breakthrough progress in the drug development of ginsenosides in recent years (**Figure 3B**). This reveals the difficulty in the research of ginsenosides and tumors. Although the exploration of the mechanism is cumulatively detailed, the translation of the results into clinically effective anti-tumor drugs still requires more research.

### Ginsenosides exert their anticancer efficacy through a variety of mechanisms

**Figure 6:**
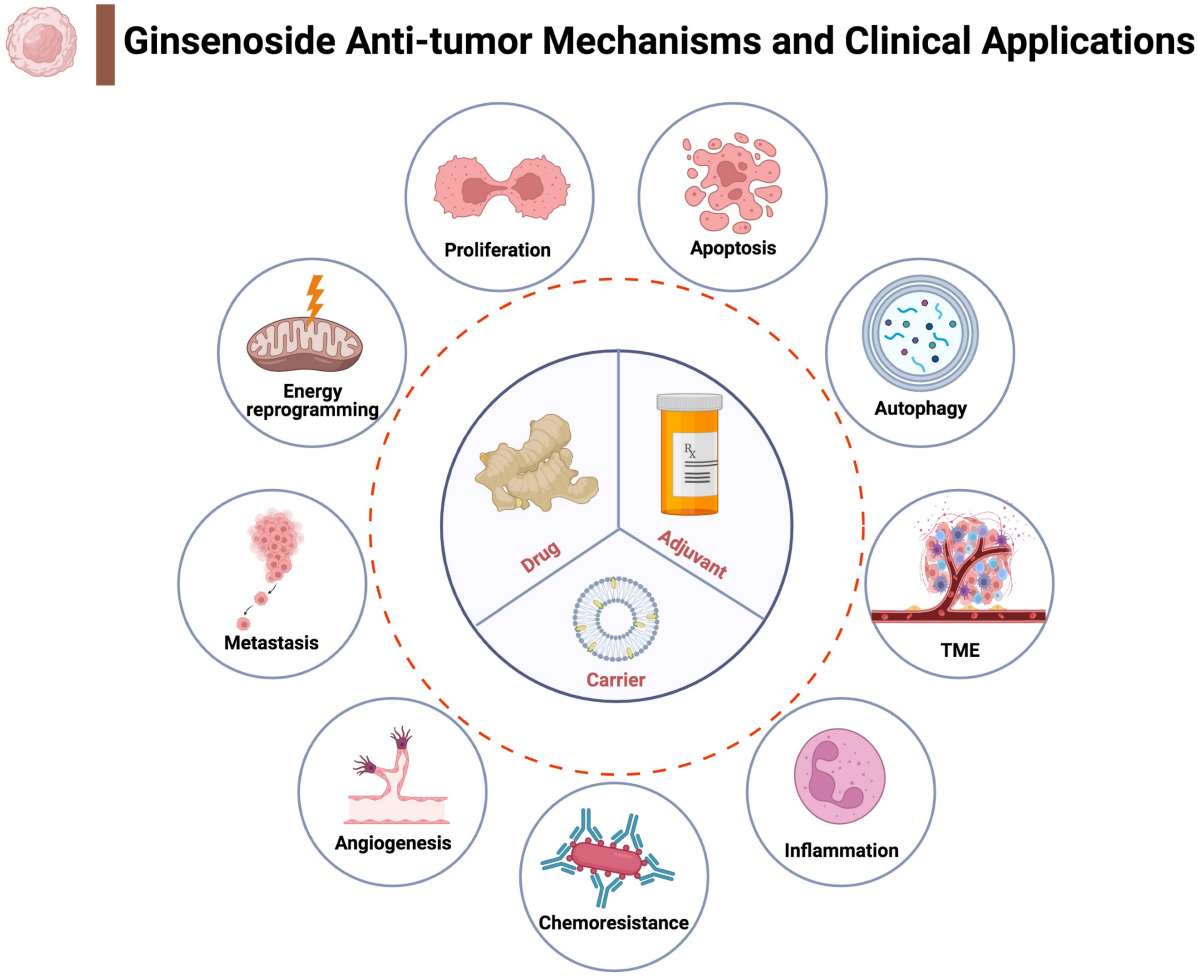
Possible anti-tumour mechanisms and clinical applications of ginsenosides. Ginsenosides could interfere with tumour growth and progression through multiple mechanisms (indicated by circles outside the dotted line). Based on the anti-tumour mechanism and chemical structure, ginsenosides have three main clinical application (indicated by circle inside the dotted line).

Cluster analysis of keywords is helpful to systematically understand the research status of this field. In the terms of pharmacological function, anti-tumor efficacy of ginsenosides and combined application in chemotherapy are research hotspots (**Figure 3A**). The potential of ginsenosides for anti-tumor therapy comes from its diverse anti-tumor mechanisms against multiple hallmarks of cancer (**Figure 6**). Through GO analysis, we found that related genes are widely distributed in the nucleus, cytoplasm and mitochondria, providing evidence for their diverse anti-tumor mechanisms (**Figure 4A**). Inhibiting tumor growth is a key point in tumor therapy. Ginsenoside Rh2 has been proved to suppress cell growth in various tumors, such as breast cancer, pancreatic cancer, prostate cancer and colorectal cancer 24-27. Telomerase activation is associated with replicative immortality which plays an important role in carcinogenesis 28. Ginsenoside Rk1 markedly inhibited telomerase activity in human hepatocellular carcinoma cells 29. The tumor-associated angiogenesis meets the need of nutrients and oxygen of cancer cells. Ginsenoside Rb1 has been proved to inhibit angiogenesis via modulating pigment epithelium-derived factor (PDEF), and may have the potential of anti-tumor agent 30. The multistep process of invasion and metastasis has been schematized as a sequence of discrete steps, leading the cause of tumor-related death. Ginsenoside Rd have been showed to inhibit the metastasis of colorectal cancer 31. Besides, Ginsenoside Rh4 could inhibit the metastasis of Lung Adenocarcinoma via suppressing JAK2/STAT3 Signaling pathway 32.

Unrestricted growth of tumor cells involves not only deregulated control of cell proliferation but also the reprogramming of energy metabolism to fuel the growth and expansion of tumor cells. Ginsenoside 20(S)-Rh2E2 downregulate of metabolic enzyme α-enolase specifically suppressing energy metabolism in lung cancer cells (LLC-1) but not normal cells. The beneficial effect and non-toxic property make ginsenosides a very promising mine of anti-tumor drugs 22. Mitochondria is the main source of cellular energy, and mitochondrial energy metabolism occurs primarily via the tricarboxylic acid (TCA) cycle and oxidative respiratory chain. Mitochondrial dysfunction may induce the accumulation of reactive oxygen species (ROS), which promote tumor formation by inducing DNA mutations and pro-oncogenic signaling pathways 33, 34. Oxidative stress is one of the research hotspots in the past five years (**Figure 3C**). Ginsenoside Re could attenuate mitochondrial dysfunction by blocking Ca2+ entry via the mitochondrial translocation of protein kinase Cδ^35^. Ginsenoside Rg2 substantially alleviate the overproduction of ROS in mice primary hepatocytes mediated by oleic acid and palmitic acid 36.

In addition to the role in energy metabolism, mitochondria could mediate apoptosis. Ginsenoside Rg3 could induced apoptosis through the mitochondrion-AIF pathway in serum-deprived cervical cancer cells 37. In another publication, ginsenoside Rg3 upregulated the pro-apoptotic protein Bax and downregulate the anti-apoptotic protein Bcl-2 to induce apoptosis of HCC cells 38. In this study, key words representing apoptosis like “cell apoptosis”, “caspase” and “bcl” were also identified, as well as the key word representing apoptosis assay like “cell viability” in the cluster of “anti-tumor efficacy and mechanism” (**Figure 3A**). Apoptosis-related terms also ranked high placing in the GO analysis of ginsenoside-related genes (**Figure 4C**). Except for mitochondria, p53 (encoded by TP53 gene) can also mediate apoptosis. TP53 is a vital tumor-suppressor gene that is mutated in numerous human cancers, and the activation of p53 in response to multiple stresses is critical for normal cells to survive and protect themselves from tumorigenesis 39. p53 could activate mitochondrial and death receptor-induced apoptotic pathways, resulting in apoptosis through the induction of caspase signaling 40. Ginsenoside Rh2 could activate the p53 pathway and consequently induce to apoptosis by increasing the levels of the anti-apoptotic protein Bax while decreasing the levels of anti-apoptosis regulator Bcl-2 10. Ginsenoside Rh4 also could activate the p53 pathway to triggers apoptosis in colorectal cancer cells 41. In gallbladder cancer cells, ginsenoside Rg3 could promotes apoptosis via the p53 pathway 42. Apoptosis determines the fate of the entire cell, and autophagy determines the fate of organelles. Although there are differences between autophagy and apoptosis in cellular morphology and central signaling pathways, the interplay between them can not to be neglected, and p53 is also critical in the interplay 43. Ginsenosides can also act on autophagy. For instance, ginsenoside Rg5 activates the MAPK signaling pathway through ROS resulting in a G2/M arrest, apoptosis and autophagy in human gastric cancer, and the inhibitory effects of Rg5 on tumor growth were verified both *in vitro* and *in vivo* xenograft model 44. Ginsenoside F2 could induce apoptosis and autophagy via modulating the phosphorylation of p53 breast cancer stem cells 45. In this study, we also found that keywords of “oxidative stress” had the strongest citation burst in the past 5 years (**Figure 3C**) and TP53 is at the center of the PPI network of ginsenoside-related genes (**Figure 5**), suggesting that the p53 signaling pathway and involved biological processes may be critical clues to deeply understand the research of ginsenosides and tumors.

Inflammation predisposes to the tumorigenesis and contribute to the all stage of tumor development. The context of a chronic inflammatory increase the risk of genetic and epigenetic damage, and conversely tumors also elicit inflammation promoting further development. In the context of a chronic inflammatory disease, inflammation exists consistently and promote tumor progression. For instance, inflammatory bowel diseases (IBD) patients have significant increased risk of colorectal cancer (CRC) 46. Patients with chronic HBV infection have increased risk of sequential development to cirrhosis and hepatocellular carcinoma (HCC) 47. Tuberculosis (TB) also may facilitate carcinogenesis 48. In the KEGG pathway analysis of this study, ginsenoside-related genes were not only highly correlated with Pathways in cancer (hsa05200), but also significantly enriched in Hepatitis B (hsa05161) and Tuberculosis (hsa05152) (**Figure 4D**). Anti-inflammatory effect is also the basis of anti-cancer properties ginsenosides. Ginsenoside Rg3 curb chronic inflammation-induced hepatic fibrosis in TAA-chronic mouse models 49. Ginsenoside Rg3 also accelerates the resolution of inflammation by inducing M2 macrophage polarization, and the pro-resolving capability of ginsenoside Rg3 was also observed in mice peritonitis model 50. Ginsenoside Rg3 directly inhibit hepatitis B virus (HBV) DNA replication and HBV-associated inflammation in human hepatocellular carcinoma cell line 51. Inflammation also has a great impact on the shaping of tumor microenvironment (TME) to offer more tumor-permissive state 52. Both tumor cells innate immune cells work together to weave a complicated network of inflammatory signals, which create a tumor-supportive microenvironment. For instance, inflammatory network and cancer-associated fibroblasts (CAFs) mutually promote tumor metastasis through angiogenesis, ECM remodeling, and epithelial-to-mesenchymal transition (EMT) 53. And interleukin 6 (IL-6) is one of the best characterized and the highest expression pro-tumorigenic cytokines in TME, which can be secreted by both CAFs and tumor cells. The regulation of tumor microenvironment is also one of the mechanisms by which ginsenosides exert their anti-tumor effects 54. Ginsenoside 20(S)-Rh2 effectively inhibited IL-6 induced signaling pathway activation (such as signal transducer and activator of transcription 3, STAT3), and IL-6 induced gene expression of matrix metalloproteinases (such as MMP-1, MMP -2, and MMP -9), resulting in inhibition of cancer cell invasion 55. EMT is one of a new focus in past five years (**Figure 3C**), and IL-6 is at the center of the PPI network of ginsenoside-related genes (**Figure 5**). Besides being a promising therapeutic candidate, we can speculate that ginsenosides can also be a promising preventive candidate for tumors.

### Ginsenosides also bring benefits in the combined application of chemotherapy

In addition to the anti-tumor effects, ginsenosides also bring benefits in the combined application of chemotherapy. The cytotoxic effect of conventional chemotherapeutic agents limits the clinical application of them. Combined application of ginsenosides helps reduce toxicity and resistance of conventional chemotherapeutic agents. When combined with chemotherapy for non-small cell lung cancer patients, ginsenoside Rg3 enhances response rate and reduces treatment-related toxicity, which prolongs overall survival rate and improving quality of life at the same time 56. Pretreatment with ginsenoside Rg5 contributes to alleviating cisplatin-induced nephrotoxicity, and the underlying mechanism may be related to anti-oxidant, anti-apoptotic and anti-inflammatory effects of ginsenoside Rg5 57. Chemoresistance is another limit for tumor therapy, and is attributed to multiple targets and pathways. Overexpression of membrane ABC transporters facilitates the efflux of chemotherapeutic agents and decrease drugs concentration at intracellular targets, which are considered as one of the major reasons resulting in chemoresistance. ABCB1 belongs to the ABC transporters family, and the activation of ABCB1 is associated with chemoresistance to numerous chemotherapeutic agents 58, 59. Ginsenoside Rh2 decrease the expression levels of ABCB1 when co-treated with oxaliplatin in colon cancer cells which is potential to reverse chemoresistance of oxaliplatin 60. Ginsenoside Rg5 could reverse the resistance of the ABCB1-overexpression MDR cancer cells to chemotherapeutic drug docetaxel (TXT) *in vitro* and *in vivo*. Different from Rh2, Rg5 activated ABCB1 ATPase and increasing the intracellular accumulation of ABCB1 substrates without altering protein expression of ABCB1^61^. Besides, ginsenosides can reverse chemoresistance by suppressing autophagy. For example, 20(S)-ginsenoside Rg3 reverse the sensitivity of icotinib-resistant human NSCLC cells to Icotinib (Epidermal growth factor receptor tyrosine kinase inhibitors) through autophagy inhibition 62. In the GO analysis, response to drug (GO:0042493) is one of the main Biological Process terms (**Figure 4C**). Those studies and our study together suggest the great potential of ginsenosides in the development of anti-tumor drugs.

### The potential of ginsenosides in nanoparticle-based drug delivery systems

Although ginsenosides have such diverse anti-tumor functions, some undesirable properties including poor aqueous solubility, membrane permeability, stability and bioavailability have limited their application in anti-tumor 5, 63. Nanomaterials help to enhance the bioavailability, targeting ability, permeability and retention effects of chemical drugs, as well as reduce toxicity in normal cells 64. Numerous novel delivery system has been used to improve efficiencies and clinical transformation of ginsenosides. Conjugating Fe@Fe_3_O_4_ nanoparticles with ginsenoside Rg3 contributes to inhibiting HCC development and metastasis to the lung 65. Ginsenoside Rb1/protopanaxadiol nanoparticles (Rb1/PPD NPs) showed high drug loading efficiency, long half-time in circulatory system, higher accumulation in tumor site and less damage to normal tissues 66. In another study, because of the structure of cholesterol-like steroidal mother nucleus, side chain of C17 and hydroxyl of C3 site, ginsenoside Rg3 can also be used as a carrier to load with docetaxel. The glucose moieties, which is the perfect ligand for glucose transporter 1 (Glut1), endow ginsenoside Rg3 the ability to target and capture CTCs in Triple-negative breast cancer 67.

This study investigated publications and related genes on ginsenosides and tumors, and discussed hot issues as well as elucidated mechanisms in this comprehensive field. However, limitations are inevitable. First, due to limitations of database and retrieval strategy, some important studies or related genes may be missed in this field. Secondly, although keywords provide the summary of publications, the preference of different authors when using keywords and the omissions caused by the highly generalized keywords will lead to bias in trend analysis. We conducted bibliometric study from 2011 to 2021. Some important and landmark studies may be omitted. In addition, only VOSviewer and CiteSpace were used for visualization and scientific analysis, the more scientific and common statistical methods like regression analysis will be explore and applied on the bibliometric research, hoping to have unexpected gains in the future. Because of the complexity of biological activities *in vivo* and the heterogeneity of tumors, further experiments are needed to confirm the mechanisms analyzed by bioinformatic method.

## Conclusion

Overall, the main objective of this study was to 1) summarize and analyze the global research trends concerning ginsenoside in tumors, 2) systematically study the antitumor mechanisms of ginsenosides. Two research centers consisting of China-South Korea-Japan and the USA-Canada respectively have made enormous contributions in this field. Current research focuses on 3 areas: “pharmacological function research”, “functional validation in animal models” and “anti-tumor efficacy and mechanism”. More precise anti-tumor mechanism research (such as inflammation and oxidative stress) will be promising in the future research of ginsenosides and tumors. The potential of ginsenosides for anti-tumor therapy comes from its diverse anti-tumor mechanisms against multiple hallmarks of cancer, among which TP53 and IL-6 as well as associated biological processes and signaling pathways may be the key points to comprehending the anti-tumor mechanism of ginsenosides. Further studies are needed to improve our understanding of this field.

## Supplementary Material

Supplementary figures.

## References

[1] Wu W, Jiao C, Li H, Ma Y, Jiao L, Liu S (2018). LC-MS based metabolic and metabonomic studies of Panax ginseng. Phytochemical analysis: PCA.

[2] Lee SM, Seo HK, Oh J, Na M (2013). Updating chemical profiling of red ginseng via the elucidation of two geometric isomers of ginsenosides Rg9 and Rg10. Food chemistry.

[3] Shibata S (2001). Chemistry and cancer preventing activities of ginseng saponins and some related triterpenoid compounds. Journal of Korean medical science.

[4] Piao X, Zhang H, Kang JP, Yang DU, Li Y, Pang S (2020). Advances in Saponin Diversity of Panax ginseng. Molecules (Basel, Switzerland).

[5] Wang H, Zheng Y, Sun Q, Zhang Z, Zhao M, Peng C (2021). Ginsenosides emerging as both bifunctional drugs and nanocarriers for enhanced antitumor therapies. Journal of nanobiotechnology.

[6] Kim YJ, Zhang D, Yang DC (2015). Biosynthesis and biotechnological production of ginsenosides. Biotechnology advances.

[7] Hanahan D, Weinberg RA (2011). Hallmarks of cancer: the next generation. Cell.

[8] Yang X, Zou J, Cai H, Huang X, Yang X, Guo D (2017). Ginsenoside Rg3 inhibits colorectal tumor growth via down-regulation of C/EBPβ/NF-κB signaling. Biomedicine & pharmacotherapy = Biomedecine & pharmacotherapie.

[9] Liu Y, Fan D (2020). The Preparation of Ginsenoside Rg5, Its Antitumor Activity against Breast Cancer Cells and Its Targeting of PI3K. Nutrients.

[10] Li B, Zhao J, Wang CZ, Searle J, He TC, Yuan CS (2011). Ginsenoside Rh2 induces apoptosis and paraptosis-like cell death in colorectal cancer cells through activation of p53. Cancer letters.

[11] Duan Z, Deng J, Dong Y, Zhu C, Li W, Fan D (2017). Anticancer effects of ginsenoside Rk3 on non-small cell lung cancer cells: *in vitro* and *in vivo*. Food & function.

[12] Yun UJ, Lee JH, Koo KH, Ye SK, Kim SY, Lee CH (2013). Lipid raft modulation by Rp1 reverses multidrug resistance via inactivating MDR-1 and Src inhibition. Biochemical pharmacology.

[13] Xing JJ, Hou JG, Ma ZN, Wang Z, Ren S, Wang YP (2019). Ginsenoside Rb3 provides protective effects against cisplatin-induced nephrotoxicity via regulation of AMPK-/mTOR-mediated autophagy and inhibition of apoptosis *in vitro* and *in vivo*. Cell proliferation.

[14] Taşkaya S, Aksoy A (2021). A bibliometric analysis of workplace incivility in nursing. Journal of nursing management.

[15] Wei Q, Shen J, Wang D, Han X, Shi J, Zhao L (2021). A bibliometric analysis of researches on flap endonuclease 1 from 2005 to 2019. BMC cancer.

[16] Mlecnik B, Galon J, Bindea G (2018). Comprehensive functional analysis of large lists of genes and proteins. Journal of proteomics.

[17] Mahmud SMH, Al-Mustanjid M, Akter F, Rahman MS, Ahmed K, Rahman MH (2021). Bioinformatics and system biology approach to identify the influences of SARS-CoV-2 infections to idiopathic pulmonary fibrosis and chronic obstructive pulmonary disease patients. Briefings in bioinformatics.

[18] van Eck NJ, Waltman L (2010). Software survey: VOSviewer, a computer program for bibliometric mapping. Scientometrics.

[19] Synnestvedt MB, Chen C, Holmes JH (2005). CiteSpace II: visualization and knowledge discovery in bibliographic d atabases. AMIA Annu Symp Proc.

[20] Elinav E, Nowarski R, Thaiss CA, Hu B, Jin C, Flavell RA (2013). Inflammation-induced cancer: crosstalk between tumours, immune cells and microorganisms. Nature reviews Cancer.

[21] Jia L, Zhao Y (2009). Current evaluation of the millennium phytomedicine-ginseng (I): etymology, pharmacognosy, phytochemistry, market and regulations. Current medicinal chemistry.

[22] Huang Q, Zhang H, Bai LP, Law BYK, Xiong H, Zhou X (2020). Novel ginsenoside derivative 20(S)-Rh2E2 suppresses tumor growth and metastasis *in vivo* and *in vitro* via intervention of cancer cell energy metabolism. Cell death & disease.

[23] Yang WZ, Hu Y, Wu WY, Ye M, Guo DA (2014). Saponins in the genus Panax L. (Araliaceae): a systematic review of their chemical diversity. Phytochemistry.

[24] Choi S, Kim TW, Singh SV (2009). Ginsenoside Rh2-mediated G1 phase cell cycle arrest in human breast cancer cells is caused by p15 Ink4B and p27 Kip1-dependent inhibition of cyclin-dependent kinases. Pharmaceutical research.

[25] Tang XP, Tang GD, Fang CY, Liang ZH, Zhang LY (2013). Effects of ginsenoside Rh2 on growth and migration of pancreatic cancer cells. World journal of gastroenterology.

[26] Gao Q, Zheng J (2018). Ginsenoside Rh2 inhibits prostate cancer cell growth through suppression of microRNA-4295 that activates CDKN1A. Cell proliferation.

[27] Yang J, Yuan D, Xing T, Su H, Zhang S, Wen J (2016). Ginsenoside Rh2 inhibiting HCT116 colon cancer cell proliferation through blocking PDZ-binding kinase/T-LAK cell-originated protein kinase. Journal of ginseng research.

[28] Luo Z, Wang W, Li F, Songyang Z, Feng X, Xin C (2019). Pan-cancer analysis identifies telomerase-associated signatures and cancer subtypes. Molecular cancer.

[29] Kim YJ, Kwon HC, Ko H, Park JH, Kim HY, Yoo JH (2008). Anti-tumor activity of the ginsenoside Rk1 in human hepatocellular carcinoma cells through inhibition of telomerase activity and induction of apoptosis. Biological & pharmaceutical bulletin.

[30] Leung KW, Cheung LW, Pon YL, Wong RN, Mak NK, Fan TP (2007). Ginsenoside Rb1 inhibits tube-like structure formation of endothelial cells by regulating pigment epithelium-derived factor through the oestrogen beta receptor. British journal of pharmacology.

[31] Phi LTH, Sari IN, Wijaya YT, Kim KS, Park K, Cho AE (2019). Ginsenoside Rd Inhibits the Metastasis of Colorectal Cancer via Epidermal Growth Factor Receptor Signaling Axis. IUBMB life.

[32] Zhang Y, Ma P, Duan Z, Liu Y, Mi Y, Fan D (2022). Ginsenoside Rh4 Suppressed Metastasis of Lung Adenocarcinoma via Inhibiting JAK2/STAT3 Signaling. International journal of molecular sciences.

[33] Gorrini C, Harris IS, Mak TW (2013). Modulation of oxidative stress as an anticancer strategy. Nature reviews Drug discovery.

[34] de Sá Junior PL, Câmara DAD, Porcacchia AS, Fonseca PMM, Jorge SD, Araldi RP (2017). The Roles of ROS in Cancer Heterogeneity and Therapy. Oxidative medicine and cellular longevity.

[35] Shin EJ, Shin SW, Nguyen TT, Park DH, Wie MB, Jang CG (2014). Ginsenoside Re rescues methamphetamine-induced oxidative damage, mitochondrial dysfunction, microglial activation, and dopaminergic degeneration by inhibiting the protein kinase Cδ gene. Molecular neurobiology.

[36] Cheng B, Gao W, Wu X, Zheng M, Yu Y, Song C (2020). Ginsenoside Rg2 Ameliorates High-Fat Diet-Induced Metabolic Disease through SIRT1. Journal of agricultural and food chemistry.

[37] Bian S, Zhao Y, Li F, Lu S, Wang S, Bai X (2019). 20(S)-Ginsenoside Rg3 Promotes HeLa Cell Apoptosis by Regulating Autophagy. Molecules (Basel, Switzerland).

[38] Jiang JW, Chen XM, Chen XH, Zheng SS (2011). Ginsenoside Rg3 inhibit hepatocellular carcinoma growth via intrinsic apoptotic pathway. World journal of gastroenterology.

[39] Huang X, Yang Z, Zhang J, Wang R, Fan J, Zhang H (2021). A Bibliometric Analysis Based on Web of Science: Current Perspectives and Potential Trends of SMAD7 in Oncology. Frontiers in cell and developmental biology.

[40] Wang X, Simpson ER, Brown KA (2015). p53: Protection against Tumor Growth beyond Effects on Cell Cycle and Apoptosis. Cancer research.

[41] Wu Q, Deng J, Fan D, Duan Z, Zhu C, Fu R (2018). Ginsenoside Rh4 induces apoptosis and autophagic cell death through activation of the ROS/JNK/p53 pathway in colorectal cancer cells. Biochemical pharmacology.

[42] Zhang F, Li M, Wu X, Hu Y, Cao Y, Wang X (2015). 20(S)-ginsenoside Rg3 promotes senescence and apoptosis in gallbladder cancer cells via the p53 pathway. Drug design, development and therapy.

[43] Mariño G, Niso-Santano M, Baehrecke EH, Kroemer G (2014). Self-consumption: the interplay of autophagy and apoptosis. Nature reviews Molecular cell biology.

[44] Liu Y, Fan D (2019). Ginsenoside Rg5 induces G2/M phase arrest, apoptosis and autophagy via regulating ROS-mediated MAPK pathways against human gastric cancer. Biochemical pharmacology.

[45] Mai TT, Moon J, Song Y, Viet PQ, Phuc PV, Lee JM (2012). Ginsenoside F2 induces apoptosis accompanied by protective autophagy in breast cancer stem cells. Cancer letters.

[46] Nadeem MS, Kumar V, Al-Abbasi FA, Kamal MA, Anwar F (2020). Risk of colorectal cancer in inflammatory bowel diseases. Seminars in cancer biology.

[47] EASL 2017 Clinical Practice Guidelines on the management of hepatitis B virus infection Journal of hepatology. 2017; 67: 370-98.

[48] Wang S, Zhou H, Zheng L, Zhu W, Zhu L, Feng D (2021). Global Trends in Research of Macrophages Associated With Acute Lung Injury Over Past 10 Years: A Bibliometric Analysis. Frontiers in immunology.

[49] Liu X, Mi X, Wang Z, Zhang M, Hou J, Jiang S (2020). Ginsenoside Rg3 promotes regression from hepatic fibrosis through reducing inflammation-mediated autophagy signaling pathway. Cell death & disease.

[50] Kang S, Park SJ, Lee AY, Huang J, Chung HY, Im DS (2018). Ginsenoside Rg(3) promotes inflammation resolution through M2 macrophage polarization. Journal of ginseng research.

[51] Hirano T (2021). IL-6 in inflammation, autoimmunity and cancer. International immunology.

[52] Greten FR, Grivennikov SI (2019). Inflammation and Cancer: Triggers, Mechanisms, and Consequences. Immunity.

[53] Marozzi M, Parnigoni A, Negri A, Viola M, Vigetti D, Passi A (2021). Inflammation, Extracellular Matrix Remodeling, and Proteostasis in Tumor Microenvironment. International journal of molecular sciences.

[54] Li M, Wang X, Wang Y, Bao S, Chang Q, Liu L (2021). Strategies for Remodeling the Tumor Microenvironment Using Active Ingredients of Ginseng-A Promising Approach for Cancer Therapy. Frontiers in pharmacology.

[55] Han S, Jeong AJ, Yang H, Bin Kang K, Lee H, Yi EH (2016). Ginsenoside 20(S)-Rh2 exerts anti-cancer activity through targeting IL-6-induced JAK2/STAT3 pathway in human colorectal cancer cells. Journal of ethnopharmacology.

[56] Xu T, Jin Z, Yuan Y, Wei H, Xu X, He S (2016). Ginsenoside Rg3 Serves as an Adjuvant Chemotherapeutic Agent and VEGF Inhibitor in the Treatment of Non-Small Cell Lung Cancer: A Meta-Analysis and Systematic Review. Evidence-based complementary and alternative medicine: eCAM.

[57] Li W, Yan MH, Liu Y, Liu Z, Wang Z, Chen C (2016). Ginsenoside Rg5 Ameliorates Cisplatin-Induced Nephrotoxicity in Mice through Inhibition of Inflammation, Oxidative Stress, and Apoptosis. Nutrients.

[58] Li W, Zhang H, Assaraf YG, Zhao K, Xu X, Xie J (2016). Overcoming ABC transporter-mediated multidrug resistance: Molecular mechanisms and novel therapeutic drug strategies. Drug resistance updates: reviews and commentaries in antimicrobial and anticancer chemotherapy.

[59] Giddings EL, Champagne DP, Wu MH, Laffin JM, Thornton TM, Valenca-Pereira F (2021). Mitochondrial ATP fuels ABC transporter-mediated drug efflux in cancer chemoresistance. Nature communications.

[60] Ma J, Gao G, Lu H, Fang D, Li L, Wei G (2019). Reversal effect of ginsenoside Rh2 on oxaliplatin-resistant colon cancer cells and its mechanism. Experimental and therapeutic medicine.

[61] Feng SL, Luo HB, Cai L, Zhang J, Wang D, Chen YJ (2020). Ginsenoside Rg5 overcomes chemotherapeutic multidrug resistance mediated by ABCB1 transporter: *in vitro* and *in vivo* study. Journal of ginseng research.

[62] Wang XJ, Zhou RJ, Zhang N, Jing Z (2019). 20(S)-ginsenoside Rg3 sensitizes human non-small cell lung cancer cells to icotinib through inhibition of autophagy. European journal of pharmacology.

[63] Zhang X, Chen S, Duan F, Liu A, Li S, Zhong W (2021). Prebiotics enhance the biotransformation and bioavailability of ginsenosides in rats by modulating gut microbiota. Journal of ginseng research.

[64] Cheng Z, Li M, Dey R, Chen Y (2021). Nanomaterials for cancer therapy: current progress and perspectives. Journal of hematology & oncology.

[65] Ren Z, Chen X, Hong L, Zhao X, Cui G, Li A (2020). Nanoparticle Conjugation of Ginsenoside Rg3 Inhibits Hepatocellular Carcinoma Development and Metastasis. Small (Weinheim an der Bergstrasse, Germany).

[66] Dai L, Zhu W, Si C, Lei J (2018). "Nano-Ginseng" for Enhanced Cytotoxicity AGAINST Cancer Cells. International journal of molecular sciences.

[67] Xia J, Ma S, Zhu X, Chen C, Zhang R, Cao Z (2022). Versatile ginsenoside Rg3 liposomes inhibit tumor metastasis by capturing circulating tumor cells and destroying metastatic niches. Science advances.

