# Supplementary material for: Ginsenosides and Tumors: A Comprehensive and Visualized Analysis of Research Hotspots and Antitumor Mechanisms
Source: J Cancer. 2024 Jan 1;15(3):671–84. doi: 10.7150/jca.88783 (PMC10777040; doi:10.7150/jca.88783)
Supplement: Supplementary file 1 — Supplementary figures. [file jcav15p0671s1.pdf]

Supplementary materials

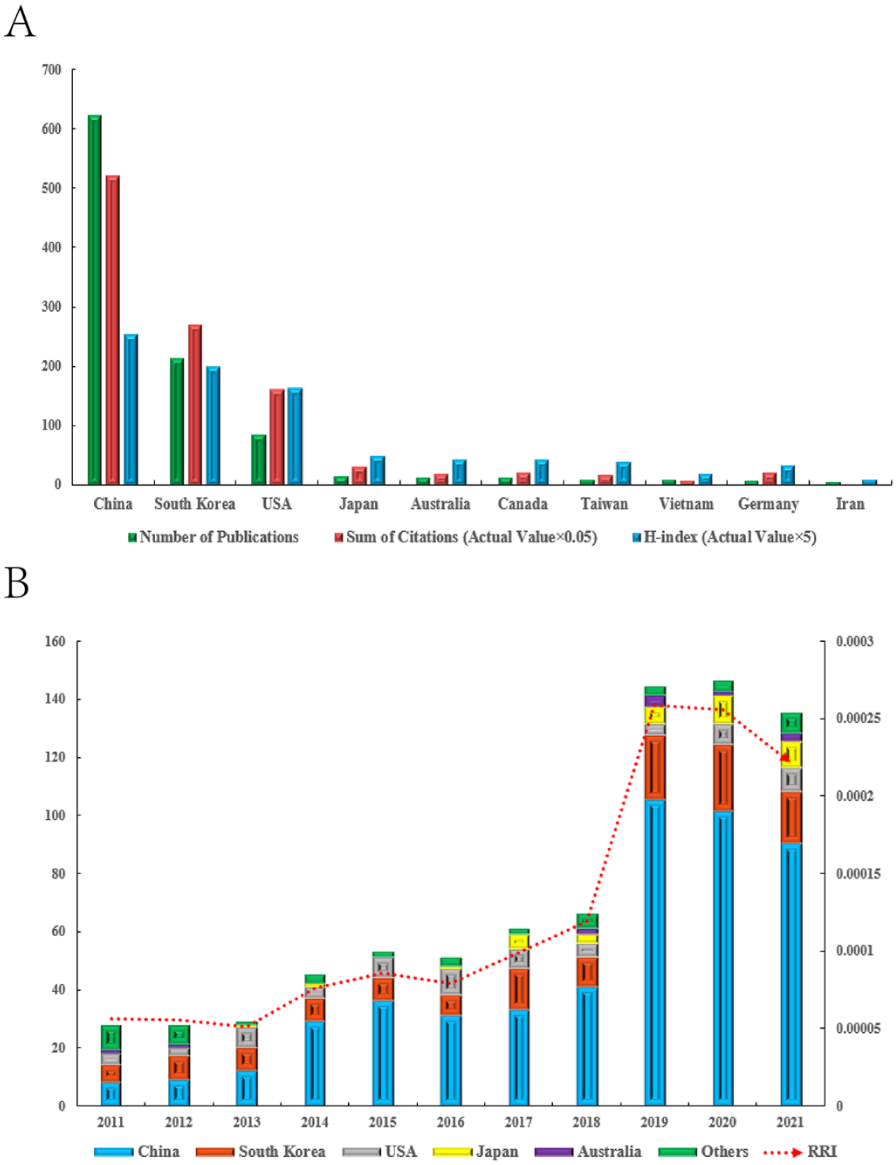

Figure S1 Articles related to ginsenoside in tumour published worldwide. (A) The number of publications, citation frequency (actual value × 0.05), and H-index (actual value × 5) in the top 10 countries or regions. (B) The annual publications worldwide and the relative research interest (RRI) for ginsenoside in tumour.

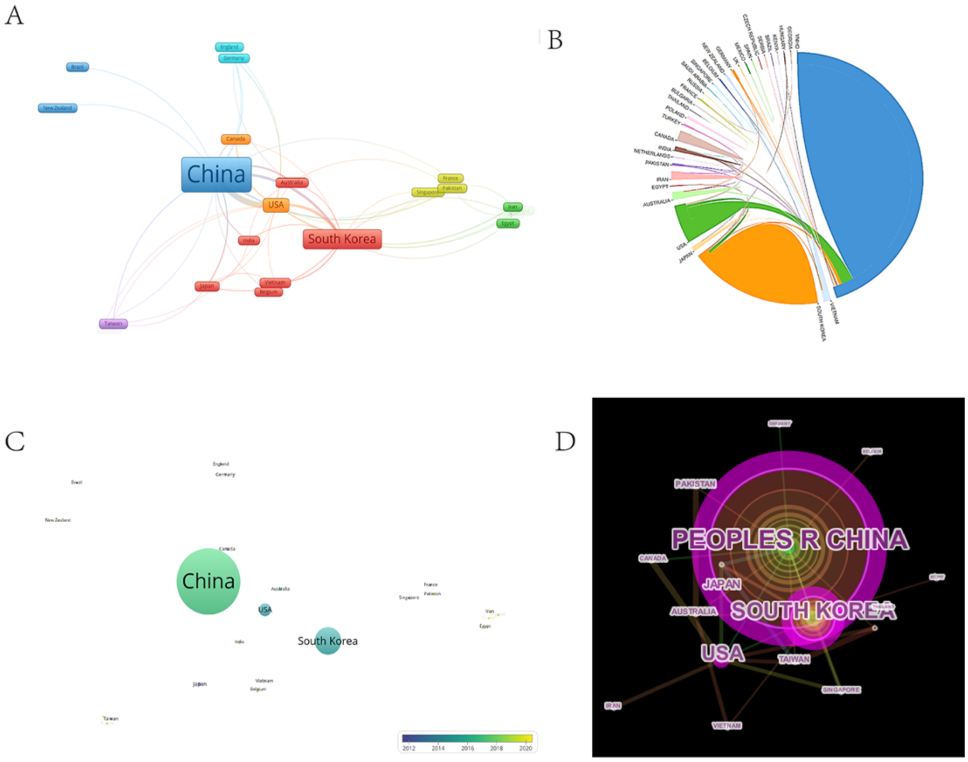

Figure S2 Analysis of collaboration between countries. (A) The collaborations between countries based on VOSviewer software. (B) Chronological order of regional distribution of publications and the collaborations between countries based on VOSviewer, and the colour of node represent the year.

A

B

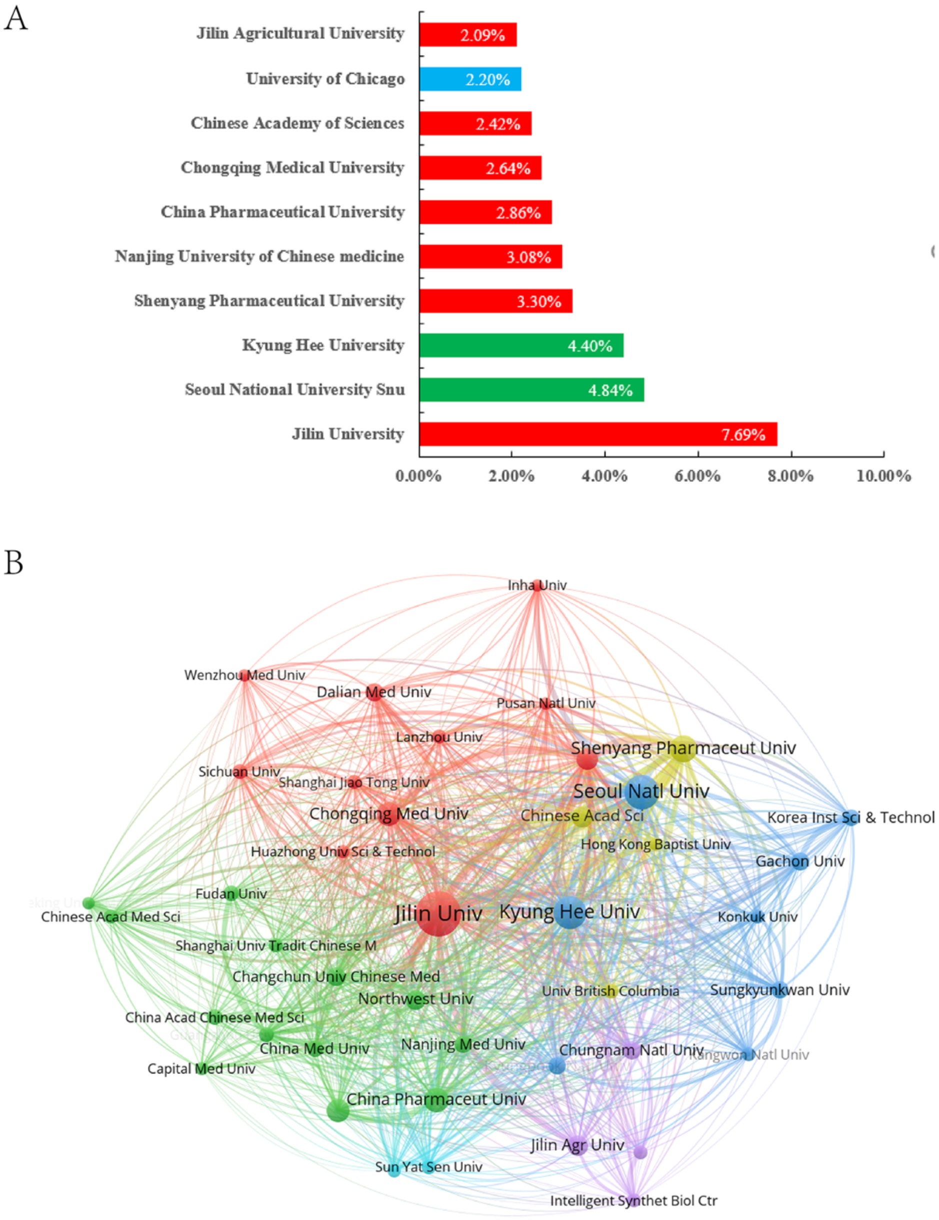

Figure S3 Articles on ginsenoside in tumour at different institutions. (A) The proportions of final-included publications of the top 10 institutions, and the colours of the bars indicate different countries. Red indicates the institution is located in China; Green indicates in South Korea; and light blue indicates in the USA. (B) Cooperation network analysis of institutions based on VOSviewer, and institutions of the same colour cooperate more closely.

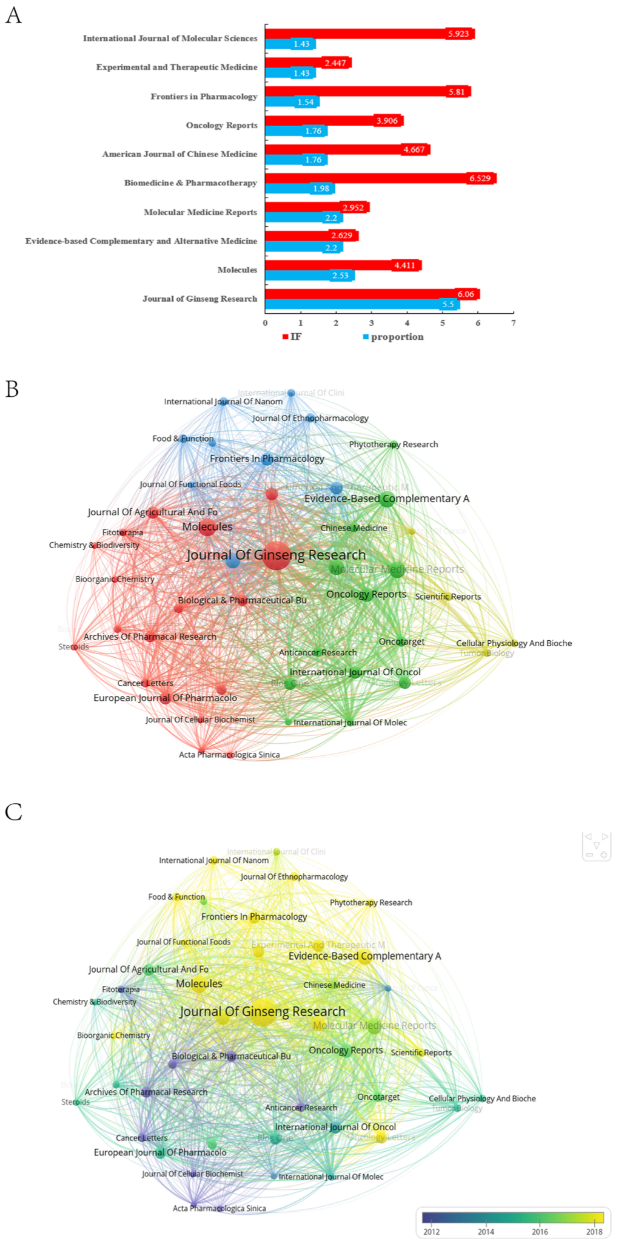

Figure S4 Articles published in different journals on ginsenoside in tumour. (A) The proportions of final-included publications and impact factors (IF) of the top 10 journals. (B) Cooperation network analysis of journal based on VOSviewer, and institutions of the same colour cooperate more closely. (C) Chronological order of journal distribution of publications based on VOSviewer, and the colour of node represent the year.
